# Analysis of Mandibular Muscle Variations Following Condylar Fractures: A Systematic Review

**DOI:** 10.3390/jcm12185925

**Published:** 2023-09-12

**Authors:** Francesco Inchingolo, Assunta Patano, Angelo Michele Inchingolo, Lilla Riccaldo, Roberta Morolla, Anna Netti, Daniela Azzollini, Alessio Danilo Inchingolo, Andrea Palermo, Alessandra Lucchese, Daniela Di Venere, Gianna Dipalma

**Affiliations:** 1 Department of Interdisciplinary Medicine, University of Bari “Aldo Moro”, 70121 Bari, Italy; assuntapatano@gmail.com (A.P.); angeloinchingolo@gmail.com (A.M.I.); robertamorolla@gmail.com (R.M.); annanetti@inwind.it (A.N.); daniela.azzollini93@gmail.com (D.A.); ad.inchingolo@libero.it (A.D.I.); daniela.divenere@uniba.it (D.D.V.); giannadipalma@tiscali.it (G.D.); 2 College of Medicine and Dentistry, Birmingham B4 6BN, UK; andrea.palermo2004@libero.it; 3 Unit of Dentistry-Orthodontics, IRCCS San Raffaele Scientific Institute, 20132 Milan, Italy; lucchese.alessandra@unisr.it; 4 Unit of Dentistry, Research Center for Oral Pathology and Implantology, IRCCS San Raffaele Scientific Institute, 20132 Milan, Italy

**Keywords:** condylar fracture, mandibular fracture, masticatory muscles, electromyography, muscles activity, bite forces, temporomandibular joint

## Abstract

This review analyzes muscle activity following mandibular condylar fracture (CF), with a focus on understanding the changes in masticatory muscles and temporomandibular joint (TMJ) functioning. Materials and Methods: The review was conducted following the preferred reporting items for systematic reviews and meta-analyses (PRISMA) guidelines. A search was performed on online databases using the keywords “masticatory muscles” AND (“mandibular fracture” OR “condylar fracture”). The eligibility criteria included clinical trials involving human intervention and focusing on muscle activity following a condylar fracture. Results: A total of 13 relevant studies were reviewed. Various studies evaluated muscle activity using clinical evaluation, bite force measurement, electromyography (EMG), magnetic sensors and radiological examinations to assess the impact of mandibular fractures on masticatory muscles. Conclusions: Mandibular condylar fractures can lead to significant changes in muscle activity, affecting mastication and TMJ functioning. EMG and computed tomography (CT) imaging play crucial roles in assessing muscle changes and adaptations following fractures, providing valuable information for treatment planning and post-fracture management. Further research is required to explore long-term outcomes and functional performance after oral motor rehabilitation in patients with facial fractures. Standardized classifications and treatment approaches may help improve the comparability of future studies in this field.

## 1. Introduction

### 1.1. Incidence and Pathogenesis

**Figure 1:**
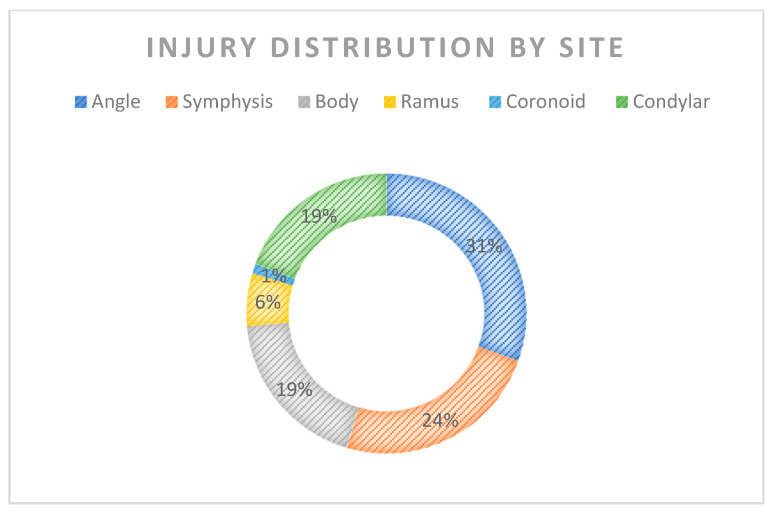
Graphic representation of injury distribution by site.

The mandible is the second most commonly fractured part of the maxillofacial skeleton after the nasal bone due to its position and prominence. The angle (27.0%), symphysis (21.3%), body (16.8%), ramus (5.4%), and coronoid (1.0%) were the most frequently injured sites, independent of mechanism [1]. Condylar fractures (CFs), instead, account for 17.5% to 52% of all mandibular fractures [2,3,4] (Figure 1).

The relative frequency of such fractures is determined by the particular morphology of the condyle, which makes it the most fragile point of the mandibular bone [5,6]. On the one hand, its slenderness tends to make it more fragile and more prone to fracture, but on the other hand, fracturing and disrupting the propulsive forces allows it to protect the glenoid cavity and skull bones [7,8]. Most are induced by indirect pressures sent to the condyle from a blow elsewhere, rather than by direct trauma. Because the coronoid process (CP) is physically protected by the zygomatico-malar complex and its accompanying muscles, isolated coronoid fractures are extremely rare [9,10]. The majority of coronoid fractures are caused by indirect blunt or penetrating trauma. Iatrogenic fractures of the CP have been reported after extractions of maxillary and mandibular third molars, sagittal split osteotomies, and cystectomies [11,12]. CF are rather common injuries; however, fractures of the CP are extremely rare, accounting for only 1% of all mandible fractures [13]. The presence of dental elements is a protective factor against traumatic impacts to the jaw, especially if the teeth are in the position of maximal intercuspation at the time of the trauma [14,15,16,17,18]. On the contrary, if some teeth are absent at the time of the trauma, for example, if the posterior sectors or the mouth is disclosed, the force is transmitted directly to the condyle with subsequent risk of fracture of the condyle [19]. In addition, the position of the muscles at the time the injury occurs is crucial in determining the direction and extent of condylar dislocation [20]. Mandibular condylar process fractures with condyle displacement cause rapid disruption of the articulating surfaces, intra-articular disc, ligaments, and muscle attachments [21]. These disturbances are followed by changes in typical maximum excursion ranges, reductions in maximum biting forces, and changes in muscle activity patterns [22,23]. CF can be either unilateral or bilateral, and a correct diagnosis based also on the muscles involved allows their proper clinical management. Among the main signs and symptoms of mandibular fracture are pain above the preauricular zone and reduced anterior opening [23,24]. The signs change depending on whether the fracture is lateral or bilateral. The unilateral CF causes a homolateral prematurity occlusion with a resulting contralateral open bite and vertical dimension loss [25,26]. Movements of laterality may appear reduced just from the contralateral side to the fractured side [27]. In most cases, bilateral CF caused by an indirect head tilt cause an open bite in addition to a loss of vertical dimension with posterior dental precontact [28].

### 1.2. Classifications of Condylar Fractures

**Figure 2:**
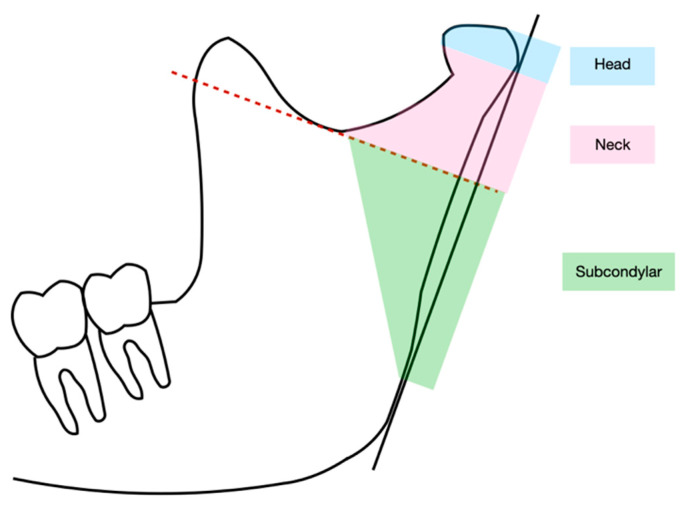
Graphic representation of the three localizations of condylar fracture. The red dotted line is the sigmoid notch line that divides the neck of the condyle from the subcondylar area.

There are numerous classifications of CF in use internationally [29,30]. As a result, the conclusions of different authors are frequently incompatible [31]. Lindahl’s classification is one of the best-known in which CF is defined according to its location [32] (Figure 2).

Thus, based on the location of the fracture they are distinguished into:

1. Head-condyle fracture: located at the level above or at the level of the joint capsule and according to its course can be defined as vertical or horizontal;
2. Condylar-neck fracture: located in the area below the head of the condyle;
3. Subcondylar fracture: located below the neck of the condyle.

### 1.3. Clinical, Instrumental Diagnosis and Treatment

Early diagnosis of CF is critical in deciding the correct treatment plan with the aim of avoiding the occurrence of subsequent complications. In case of suspected mandibular fracture, very often as a result of sustained trauma, it is necessary to proceed with an objective clinical examination based on palpation of the area, both intra-oral and extra-oral inspection, and assessment of joint function [33,34]. Radiographic investigations are then required for diagnostic confirmation. For the study of the TMJ, the specific examinations are directly computed tomography (CT) in axial and coronal projection and orthopantomography (OPT) [35]. In the diagnostic phase, CT with tridimensional reconstructions can also be of great help [36]. To assess the function of the masticatory muscles, electromyography (EMG) can be performed, which uses skin electrodes to record the activity of the muscle fibers both in activity and at rest [37,38].

For many years, conservative therapy was considered the gold standard for the treatment of the mandibular CF [39]. In the latest period, however, many surgeons tend to prefer surgical treatment as the best solution, probably because of the new technologies introduced [40,41,42]. In fact, the surgical procedure achieves fracture reduction, which together with internal fixation, allows a good anatomical repositioning to be achieved [43]. Usually, surgical treatment is chosen in cases where conservative treatment fails to achieve proper “restitutio ad-integrum” of the fracture site [44,45].

Muscular therapy following temporomandibular disorders caused by condyle fracture is critical for proper masticatory function recovery. It tries to restore muscle balance and enhance joint mobility with focused workouts, manual treatment, and specialized equipment [46]. The objective is to alleviate discomfort, restore normal biomechanics, and avoid muscle compensation. Major treatments for the management of pain caused by temporomandibular disorders include physical therapy, drug therapy, laser therapy, occlusal therapy, oxygen–ozone therapy, extracorporeal shock wave therapy, and transcutaneous electrical stimulation [47].

The aim of this review is to analyze muscle activity following mandibular CF.

## 2. Materials and Methods

### 2.1. Protocol and Registration

This study was carried out in accordance with the Preferred Reporting Items for Systematic Reviews and Meta-Analyses (PRISMA) guidelines and submitted to PROSPERO (International Prospective Register of Reviews) with 448,110 [48].

### 2.2. Search Processing

The search was conducted on 13 July 2023 on the PubMed, Scopus, and Web of Science databases without the inclusion of any time interval. The search approach included the following Boolean keywords: “masticatory muscles” AND (“mandibular fracture” OR “condylar fracture”). These keywords were chosen because they most accurately reflected the aim of our investigation, which was to find out more about the activity and function of the masticatory muscles following a unilateral or bilateral mandibular CF.

### 2.3. Eligibility Criteria and Study Selection

The two steps of the selection process were the appraisal of the title and abstract and the complete text. Any article that fit the following requirements was taken into consideration: (a) clinical trials including human intervention; (b) muscle activity following a condylar fracture; (c) free full text. Publications (such as meta-analyses, research methods, conference papers, in vitro or animal experiments) that lacked original data were not included. Titles and abstracts from the preliminary search were retrieved and evaluated for relevance. Full articles from pertinent research were acquired for further analysis. The retrieved studies were assessed for inclusion using the aforementioned criteria by two different reviewers (R.M. and A.P.).

### 2.4. Data Processing

R.M. and A.P., the two reviewers, independently evaluated the quality of the studies, based on selection criteria after performing a database search to extrapolate the findings. In order to use with Zotero, the chosen articles were downloaded in the 6.0.15 version. A senior reviewer (F.I.) was consulted in order to address any disagreements between the two reviewers.

### 2.5. PICOS Requirements

The PICOS (population, intervention, comparison, outcome, study design) criteria, which are used in this evaluation, encompass population, intervention, comparison, outcomes, and study design (Table 1).

### 2.6. Quality Assessment

The quality of the included papers was assessed by two reviewers, RF and EI, using the reputable Cochrane risk-of-bias assessment for randomized trials (RoB 2). The following six areas of possible bias are evaluated by this tool: random sequence generation, allocation concealment, participant and staff blinding, outcome assessment blinding, inadequate outcome data, and selective reporting. A third reviewer (FI) was consulted in the event of a disagreement until an agreement was reached.

## 3. Results

### 3.1. Selection and Characteristics of the Study

**Figure 3:**
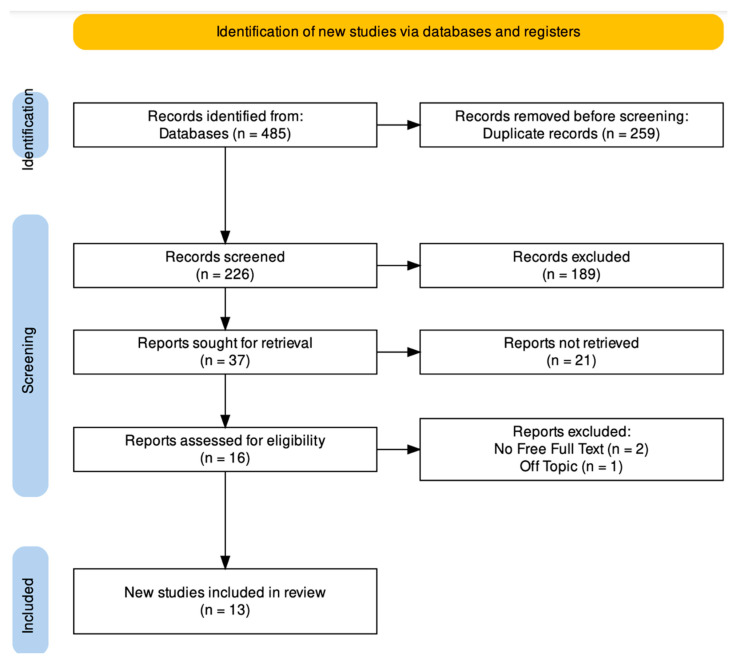
PRISMA ScR flowchart diagram of the inclusion process.

A total of 485 publications were found in the online database (PubMed n = 211, Scopus n = 199, and Web of Science n = 75); no papers were found using a manual search. After 259 duplicate studies were removed, 226 studies were evaluated by looking at the title and abstract. From here, 37 records were chosen out of 189 items that failed to fulfill the requirements for inclusion. Subsequently, 21 non-retrieved records were excluded. There were 16 reports evaluated for eligibility, and three reports were removed. In the end, 13 studies were reviewed for the qualitative analysis. The selection process and the summary of selected records are shown in Figure 3. The study characteristics are summarized in Table 2.

The following review analyzes a total of 13 papers including four case control (30%), four observational studies (30%), three prospective cohort studies (24%), and two retrospective studies (16%). Among the analyzed studies, three deal exclusively with bilateral fractures, five deal exclusively with unilateral fractures, and the last five consider both unilateral and bilateral fractures.

### 3.2. Quality Assessment and Risk of Bias

**Figure 4:**
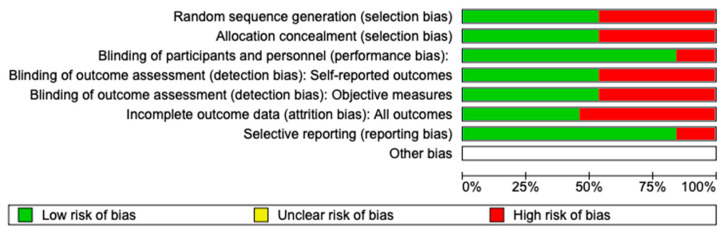

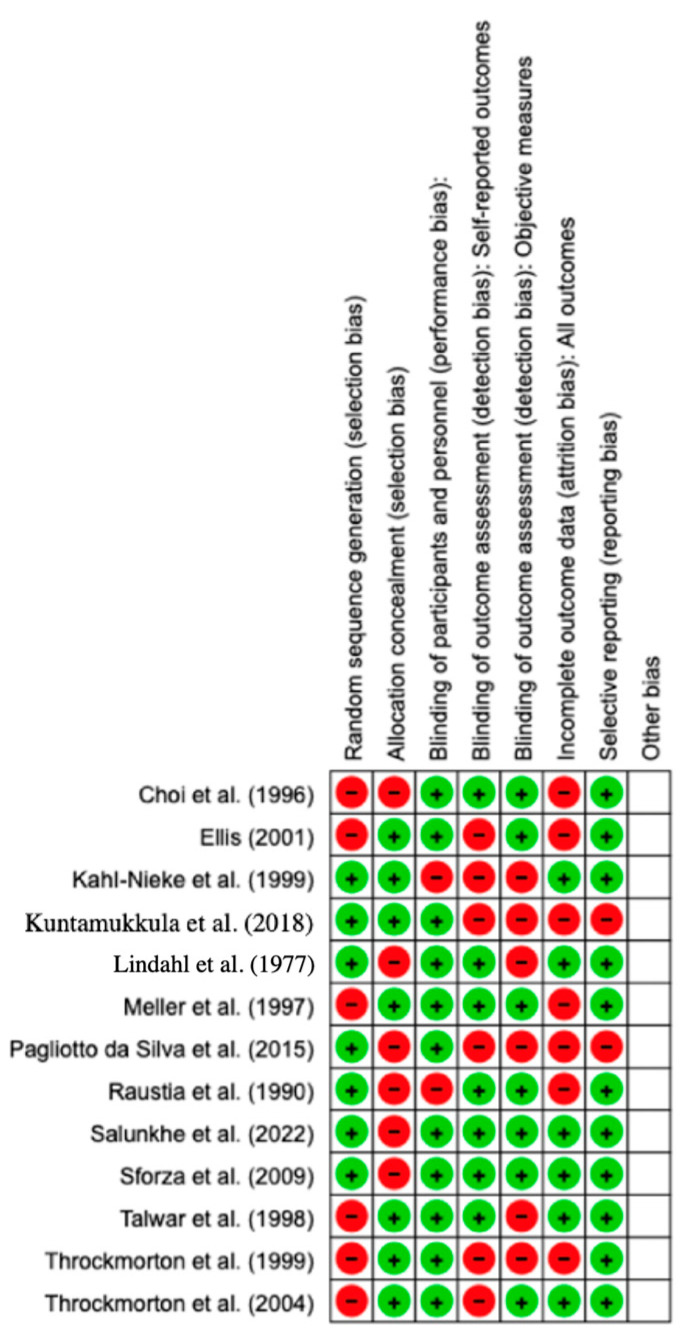
Risk of bias; red indicates high risk, and green indicates low risk of bias. Choi et al. [51]; Ellis et al [21]; Kahl-Nieke et al. [52]; Kuntamukkula et al. [53]; Lindahl et al. [32]; Meller et al. [58]; Pagliotto da Silva et al. [59]; Raustia et al. [54]; Salunkhe et al. [55]; Sforza et al. [56]; Talwar et al. [49]; Throckmorton et al. [50]; Throckmorton et al. [57].

The risk of bias in the included studies is reported in Figure 4. Regarding the randomization process, 50% of studies present a high risk of bias and allocation concealment. All other studies ensure a low risk of bias: 75% of studies exclude a performance; 50% of studies confirm an increased risk of detection bias (self-reported outcome); and 50% of the included studies present a low detection bias (objective measures) (Figure 4). Two studies ensure a low risk regarding attrition and reporting bias.

## 4. Discussion

Following mandibular condylar fractures, alterations in masticatory muscles occur, impacting temporomandibular joint function. Assessing these changes requires comprehensive methods. Clinical palpation, bite force analysis, electromyography, CT scans, and magnetic jaw tracking are employed to scrutinize muscular alterations. These techniques offer insights into muscle functionality, symmetry, and coordination post-fracture, aiding a comprehensive understanding of post-traumatic masticatory muscle adaptations and their implications on mandibular function.

### 4.1. Clinical Palpation

Lindahl’s study proposed to understand the recovery process and the potential development of dysfunction in the masticatory system following CF. Examiners performed a comprehensive clinical examination, combined with radiographic evaluations, on a group of 67 individuals of different age groups [32]. During clinical examinations, several parameters related to muscle function were recorded, such as palpability of the condylar head to determine the presence of any dislocations, measurement of mandibular movements (maximum opening, laterotrusion and protrusion), and detection of functional symmetry [32]. The results provide valuable information about the long-term consequences of CF and that there are significant variations among different age groups. Indeed, in children, most fractures heal without causing significant masticatory dysfunction. In adolescents, dysfunction is more frequent but usually less severe than in adults. The latter, however, show a higher incidence of symptoms of masticatory dysfunction, potentially related to persistent dislocation of the condylar fragment [32].

### 4.2. Bite Force

Mastication is a crucial function of the stomatognathic system, and chewing force is an important parameter for assessing the restoration of function after surgery.

Talwar’s study analyzes the effects of bilateral fractures of condylar processes on chewing force. Through the analysis of data from cephalograms and biomechanical studies, significant morphological and biomechanical changes were found in patients with CF [49]. A decrease in bite force was observed in the first few months after the fracture, with a gradual recovery over time. This decrease in force appears to be related to complex adaptations of the masticatory system, including changes in craniofacial morphology and muscle activity [49]. Indeed, the results showed a hyperdivergent craniofacial morphology in bilateral fractures, with lower posterior facial height, higher mandibular and genial angles. In addition, temporalis muscle activity was found to be more involved in chewing in patients with CF, suggesting an adaptation strategy to reduce the load on the fractured joints [49].

In an American study, the authors investigated whether chewing force in patients with unilateral fractures of the condylar process of the mandible varied according to the type of restorative treatment whether with open or closed techniques. Data from 155 patients with unilateral fractures of the condylar process of the mandible treated with open or closed techniques were analyzed [21]. Maximum clamping force measurements were made using a force transducer. Despite the different treatment approaches, both groups of patients show a similar ability to generate occlusal forces. However, neuromuscular adaptations in muscle recruitment during chewing on the side opposite the fracture are observed, indicating a strategy to protect the injury site [21]. In summary, patients adjust to reduce the load on the fracture, but chewing ability does not vary significantly between the two treatment approaches [21].

In contrast, Kuntamukkula’s study evaluates the dynamic stability of the TMJ in patients with unilateral condyle fractures undergoing reduction surgery and open internal fixation. During the six-month recovery period, bite force was evaluated both statically and dynamically on both sides of the mandible [53]. The results indicate that although the maximum bite force is similar on both sides, the mean functional bite force is significantly higher on the unoperated side, suggesting lower masticatory efficiency on the injured side [53]. Neuromuscular adaptations and early movements of the condyle-disc complex on the operated side were also observed, indicating that despite restoration of TMJ anatomy with open treatment, dynamic stability may take more than six months to be achieved [53].

Salunkhe et al., also investigated significant changes in masticatory loads in patients with mandibular condyle fractures undergoing reduction surgery and open internal fixation. The results showed that, initially, the bite force in the molar region was significantly reduced in the first postoperative week, but gradually approached the levels of the control group by the ninth postoperative week [55]. This suggests that the healing and restoration of normal architecture led to a gradual and modest increase in biting forces. It was also observed that unilateral fractures of the mandibular condyle had a greater impact on biting force than bilateral fractures. The evolution of stability and masticatory function took a longer period in the case of bilateral fractures [55]. These results underscore the importance of careful evaluation and an adequate recovery period after surgery to ensure complete restoration of masticatory function in patients with mandibular condyle fractures [55].

Fractures of the mandibular condyle may temporarily affect chewing strength, but with proper surgical treatment and recovery period, chewing ability tends to improve over time.

### 4.3. Electromiography

Sforza’s study aims to quantitatively assess the percentage contribution of rotation and translation movements of the mandible during maximum mouth opening in patients treated for CF [56]. The researchers used EMG to measure the activity of masticatory and neck muscles during maximum voluntary teeth clenching. They also calculated different EMG indices to develop simpler estimations of TMJ functioning [56].

The results indicated that patients showed altered patterns of mandibular motion during mouth opening, with a larger percentage of rotation and reduced vertical displacement compared to healthy individuals. EMG indices in patients also differed from those in the healthy subjects, indicating potential functional adaptations following the fractures [56]. Overall, the study provides valuable insights into the changes in masticatory muscles after mandibular fractures, especially in relation to TMJ functioning, which can aid in the treatment and management of such injuries [56].

Hjorth’s study also evaluates parameters recorded through EMG: it reports the results of a longitudinal study conducted on patients with unilateral fractures of the mandibular condyle [58]. The aim of the study was to examine the effects of these fractures on the activity of the temporal and masseter muscles up to 6–12 months after the trauma, using EMG to record muscle changes [58].

During observation, the level of muscle activity at rest (postural) did not change significantly. However, there was a significant increase in muscle activity during maximal contraction of the contralateral temporalis and masseter muscles (on the side opposite the fracture) from the time immediately after fracture treatment (T0) until 6–12 months later (T2). Furthermore, during natural mastication, the maximal activity of the anterior temporalis muscle increased significantly over time [58].

During unilateral mastication (UM, performed on one side only), a significant increase in activity was noted in the anterior temporalis and contralateral masseter muscles from T0 to T2. Furthermore, muscle contractions became shorter and stronger in all muscles during UM, except for the ipsilateral masseter (on the same side as the fracture), which had a significant increase in activity only between the first and second examinations [58].

In summary, EMG revealed significant changes in muscle activity in patients with unilateral mandibular condyle fractures. Increases in the maximal muscle activity of the contralateral temporalis and masseter muscle were observed during contraction and natural chewing [58]. Furthermore, during UM, stronger and shorter contractions occurred in all muscles involved, except in the ipsilateral masseter. These results indicate a muscular adaptation in the course of time after the fracture and suggest a possible role of the suppression reflex in the muscle of the side of the fracture as a consequence of the trauma [58].

Pagliotto da Silva aimed to investigate the oral motor function in patients with facial fractures [59].

The study included 38 adult patients with facial fractures, divided into two groups based on the treatment received: Group 1 (G1) received open reduction of facial fractures, and Group 2 (G2) received closed reduction with maxillomandibular fixation. A control group of 19 healthy volunteers was also recruited [59].

The participants underwent oral motor clinical assessment, mandibular range of movement measurements, and surface electromyography (sEMG) evaluation of the anterior temporal and masseter muscles. The data were analyzed using non-parametric tests.

The results showed that patients with facial fractures had poorer performance in oral motor functions, including swallowing and mastication, compared to the control group. Both G1 and G2 exhibited a limited mandibular range of motion, particularly in maximal incisor distance. The electromyographic assessment revealed that both groups of patients with facial fractures had lower overall muscle activity compared to the control group [59].

However, there were no significant differences in oral motor function or muscle activity between G1 and G2, suggesting that the severity of facial fractures did not influence muscle function and performance four months after fracture correction.

Overall, the study indicates that patients with facial fractures experience deficits in oral motor function, but the type of fracture treatment (open or closed reduction) did not significantly affect the outcomes of muscle function and performance after four months of treatment. The electromyographic evaluation of the anterior and posterior heads of the temporalis, masseter and sternocleidomastoid muscles in bicondylar fractures showed a lower activity of the masseters than that of the temporals during the closing phase, especially on the working side, the opposite of what normally happens [57]. During the occlusal phase, on the other hand, the muscle force is reduced compared to healthy patients, partly because the reduced activity of the lateral pterygoid muscle tends to overload the balancing condyle [57].

### 4.4. CT Scan

A practical tool for evaluating soft tissue changes in the nervous system after a CF that is not visible on standard radiographs is CT. It has been used to determine the disc’s position and assess how well it is functioning. It has also been shown to be superior to traditional radiography in terms of detecting subtle changes in the condylar head and the mandibular fossa [60,61,62]. Additionally, CT has been useful in examining the skeletal muscles to detect muscular atrophy, pseudohypertrophy, denervation atrophy and hypertrophy [62,63,64].

A CT scan analysis of the masseter, temporalis and lateral pterygoid muscle showed that the first two muscles on the fractured side have a similar density to those on the non-fractured side, although slightly lower. The only statistically significant difference is related to the lower density of the lateral pterygoid muscle on the fractured side compared to the non-fractured side, considering that the lateral pterygoid has absolutely less density than the rest of the other muscles. In some cases, the muscle shows a reduction in density in direct proportion to the passage of time [54].

Kahl-Nieke et al. analysed the changes undergone by the lateral pterygoid muscle in 19 child patients with a unilateral fracture during and after treatment with functional devices that promote condylar remodeling. The device was worn for approximately nine months and was intended to promote condyle formation with resorption of the fractured fragment. In the follow-up in more than 70% of the patients, there was a reduction in the muscle mass of the lateral pterygoid in the range of 13 to 69%. The extent of the mass reduction is directly proportional to the depth and severity of the fracture: deep fractures and fractures with complete dislocation of the disc suffered the greatest mass reduction [52].

In patients with bilateral CF due to muscle contraction, there is an open-bite and retroposed position of the mandible. After 10 days of intermaxillary fixation, months of jaw exercises, muscle training, and mandibular manipulation, the open bite was removed. Masticatory muscle adaptation allows for the restoration of occlusion and function [51].

### 4.5. Magnetic Jaw Track Device

Following a mandibular fracture, in addition to the alteration of the anatomy of the TMJ the function also changes: the masticatory cycles are altered, especially the changes that the two ends of the lateral pterygoid muscle undergo [65,66].

To assess the masticatory cycles, measurements were taken during the chewing of a gummy bear after the insertion of a small magnet at the level of the lower incisors, the patient’s face with the frankfurter plane parallel to the floor. A magnetic sensor array (Sirognathograph; Siemens Corp, Bensheim, Germany) was attached to the patient’s head to monitor the activity of the magnet. All these devices are connected to a computer that reports what occurs in the three planes of space [54,57,67,68,69,70,71,72,73].

In the case of bicondylar fractures, the muscles affected are the two ends of the lateral pterygoid, which makes the protrusion movement difficult since it fits over the head and neck of the condyle. The other muscles are not particularly affected [50].

The study proposed by Throckmorton hypothesizes that in unilateral CF treated with the closed technique, the alterations of the masticatory cycles persist even after bone healing, unlike what happens with the open technique [57].

The patients were divided into two groups: the first group was treated surgically, using a retromandibular approach for surgery in the condylar area. In these patients, plates were fixed without compression using at least two screws of 2 mm length. The second group included patients who did not receive any surgical treatment of the condyle but used Class II elastics to guide the mandible into correct occlusion and favor pseudocondylar formation. Patients in both groups followed the same physiotherapy exercises and the use of rubber bands to guide correct occlusion [57].

These patients were treated either surgically or in a combined manner (one side was treated with surgery and the other with a conservative approach [50].

At a follow-up of two years, the inferior excursion remains reduced compared to the control of healthy patients [57]. The excursion is lower if the fracture is bicondylar [50].

The posterior opening range in patients treated with both techniques is between 4.7 and 5.5 mm after six weeks to one year; in patients with a bicondylar fracture it is even wider. In the control group, on the other hand, the maximum posterior excursion is 4 mm. In the control two years after the end of treatment, however, the values tend to decrease and return to a normal range. Additionally, in the open group, condylar mobility on the fractured side was lower than that on the non-fractured side six months after the fracture, whereas in the closed group, condylar mobility on both sides was essentially comparable. Even while these variations were not statistically significant, they do at least imply that the open group’s condylar mobility may have been slightly higher after six weeks [50,57].

A significant difference was evident one year post-treatment with better stabilization in the group treated with the conservative technique than in the group treated with the open technique: lateral excursion on the fractured side returns to the normal range as early as six months, while in surgically treated patients it takes at least one year [57].

The more used measures of masticatory function (duration and excursive ranges) are not significantly affected by surgical treatment of unilateral condylar process fractures. On the side opposite the fracture, however, surgical treatment better normalizes opening incisor channels during mastication. Similar to the opening phases, the patients’ reduced excursion toward the working side during the fast-close phase is consistent with the inhibition of normal translation of the balancing side condyle after bilateral condylar process fractures [50].

The review has several limitations:

1. Heterogeneous study designs: The included studies vary in design, such as case-control, observational, retrospective, and prospective studies. Combining data from different study designs may introduce heterogeneity and affect the validity of the review’s conclusions.
2. Limited sample sizes: Some of the included studies have small sample sizes, which could limit the statistical power and generalizability of the findings.
3. Lack of quality assessment: The review does not mention whether a quality assessment of the included studies was conducted. Evaluating the methodological quality of the studies is essential to assess the overall reliability of the evidence.
4. Limited scope of analysis: The review mainly focuses on muscle activity following mandibular CF, but it does not discuss other potential outcomes or complications related to these fractures, such as pain, joint function, or psychosocial impact. A more comprehensive analysis of the implications of these fractures would provide a more robust understanding of the topic.

## 5. Conclusions

In conclusion, the systematic review of literature has revealed a multifaceted landscape of evidence regarding the impact of condylar fractures on the masticatory system and the subsequent recovery processes. Clinical palpation studies underscore the age-dependent variations in the consequences of condylar fractures, with children generally experiencing milder dysfunction compared to adolescents and adults, potentially related to persistent condylar fragment dislocation. Bite-force analyses elucidate the dynamic adaptations of the masticatory system post-fracture, with reductions in force initially observed and subsequent adjustments to protect the injured site. Electromyography studies offer insights into muscle activity changes, revealing muscular adaptations and functional alterations, particularly in patients with unilateral fractures. CT scans provide valuable insights into soft tissue changes and muscular atrophy, emphasizing the importance of long-term evaluation. Magnetic jaw track device studies shed light on altered masticatory cycles following fractures, with differences observed between surgical and non-surgical treatments.

Overall, these findings underscore the complexity of the masticatory system’s response to condylar fractures and the importance of tailored treatment approaches and long-term monitoring for optimal recovery. Further research is needed to enhance our understanding of these processes and to guide more effective clinical management strategies for patients with mandibular condylar fractures.

## Figures and Tables

**Table 1:** PICOS criteria.

**Criteria**	Application in the present study
**Population**	Subjects suffered CF.
**Intervention**	Surgical or conservative treatment of condylar mandibular fracture (unilateral or bilateral).
**Comparisons**	Comparison of different methods of recording muscle activity (EMG, CT scans, clinical palpation).
**Outcomes**	Changes in masticatory muscle activity following condylar fracture detected by: clinical palpation, bite force, electromyography, CT scan, and magnetic jaw track device.
**Study design**	Case control studies, observational studies, prospective cohort studies, retrospective studies.

**Table 2:** Characteristics of the studies included in the qualitative analysis.

Author	Study Design	Number of Patients/Gender	Country	Type of Fracture (Unilateral/Bilateral)	Matherial and Methods	Outcomes
1-Talwar et al. (1998) [49]	Case-control	22 (15 M–7 F)	USA	Bilateral	Compared open reduction (n = 6), closed therapy (n = 14), or a combination (n = 2) with 22 controls	Reduced posterior face height and moment arm length in masseter and pterygoid muscles. Different temporalis muscle orientations. Restricted movement during the procedure. Reduced bite forces in patients. Temporalis muscle used more during maximal biting
2-Ellis (2001) [21]	Observational study	155 (127 M–28 F)	USA	Unilateral	Bite force of masseter muscles at intervals	No significant differences in bite forces between treatment groups. Improved bite force over time
3-Throckmorton et al. (1999) [50]	Case-control	22 (15 M–7 F)	USA	Bilateral	Examined patients and controls over time. Recorded incisor movements and muscle activity.	Reduced anterior translation and lateral excursion due to anatomical disruption and poor lateral pterygoid function. Reduced muscle activity during closure stages. Most individuals resumed regular eating within a year
4-Choi et al. (1996) [51]	Observational study	10 M	Korea	Bilateral	Patients with various symptoms and CT scans showing bilateral CF	Jaw exercises and mandibular manipulation eliminated open bite after IME release. Restoration of occlusion and function through masticatory muscle adaptation
5-Lindahl et al. (1977) [32]	Observational study	67 patients	Sweden	Bilateral and unilateral	Radiographic and clinical examination at intervals	Maximal opening returned to normal in most individuals after two years Children showed fewer persistent joint and muscle complaints than adults
6-KahI-Nieke et al. (1999) [52]	Observational study	19 patients (9 F and 10 M)	Germany	Unilateral	Analyzed post-traumatic and post-therapeutic state of soft tissue in children with CF	Functional restoration was good or very good after an average of 5 years. Lateral pterygoid muscle diminished in nearly two-thirds of patients. Changes in volume between healthy and damaged sides
7-Kuntamukkula et al. (2018) [53]	Prospective cohort study	30 patients	India	Unilateral	Evaluated TMJ dynamics and muscle EMG	TMJ abnormalities persisted six months after the treatment of CF with open reduction and internal fixation. Long-term studies needed for accurate timeline
8-Raustia et al. (1990) [54]	Restrospective study	17 patients	Finland	Unilateral/bilateral	Evaluated muscle density with CT after mean period of 33 months	No significant differences in muscle density, but lateral pterygoid muscle is smaller on the fractured side. Difference increased with time
9-Salunkhe et al. (2022) [55]	Case-control study	30 patients	India	Unilateral/bilateral	Divided into groups based on fracture type and compared masticatory forces with a control group	Temporary reduction in chewing force observed in unilateral fracture cases, gradually restored over time. Bilateral fractures took longer to restore the chewing force
10-Sforza et al. (2009) [56]	Case-control study	9 patients (8 M, 1 F)	Italy	3 unilateral and 6 bilateral	Evaluated mandibular movements and EMG activity during clenching	Patients had a significantly higher percentage of rotational movement. Changes in mandibular movements and EMG indices compared to healthy subjects
11-Throckmorton et al. (2004) [57]	Prospective study	81 male patients	USA	Unilateral	Recorded chewing cycles with sensor array during mastication on both sides. Surgical correction normalized incisor pathways on the opposite side	Surgical correction better normalizes opening incisor pathways during mastication on the side opposite the fracture.
12-Meller et al. (1997) [58]	Retrospective study	9 adult men	Denmark	Unilateral	Evaluated muscle contractions during chewing before and after treatment.	Significant increase in muscle contractions on impaired joint side during chewing after treatment.
13-Pagliotto da Silva et al. (2016) [59]	Prospective cohort study	26 adults, both gender.	Brasil	Unilateral and bilateral	Divided into groups based on treatment. Assessed orofacial myofunctional system and mandibular range of motion	Surgical open reduction showed better symmetry in masseter muscle activation compared to closed reduction treatment

## Data Availability

Not applicable.

## Abbreviations

CF	Condylar fracture
CP	Coronoid process
CT	Computed tomography
EMG	Electromyography
F	Female
M	Male
OPT	Ortopantomography
sEMG	Surface electromyography
TMJ	Temporomandibular joint
UM	Unilateral mastication
